# Efficient HIV-1 in vitro reverse transcription: optimal capsid stability is required

**DOI:** 10.1038/s41392-020-00458-3

**Published:** 2021-01-12

**Authors:** Ryan C. Burdick, Vinay K. Pathak

**Affiliations:** Viral Mutation Section, HIV Dynamics and Replication Program, Center for Cancer Research, National Cancer Institute at Frederick, Frederick, MD 21702 USA

**Keywords:** Structural biology, Vaccines

Recently, Christensen et al. reconstituted HIV-1 reverse transcription and integration in a cell-free system and found that viral double-stranded DNA that is capable of integration into a target plasmid DNA was efficiently synthesized within viral cores, indicating that the capsid protein shell plays an important role in viral genome replication.^1^

HIV-1 reverse transcriptase (RT) copies the viral genomic RNA into a double-stranded DNA, and HIV-1 integrase (IN) inserts the viral DNA into the host genome to form a provirus. Both RT and IN have been successfully targeted with potent antiretroviral drugs to inhibit these enzymes and treat patients infected with HIV-1, the causative agent of acquired immunodeficiency syndrome (AIDS). Despite decades of research, the molecular mechanisms of reverse transcription and integration in vivo are not fully characterized, in part because cell-free assays that efficiently and faithfully recapitulate the processes in the context of a virion have not been available.

**Fig. 1:**
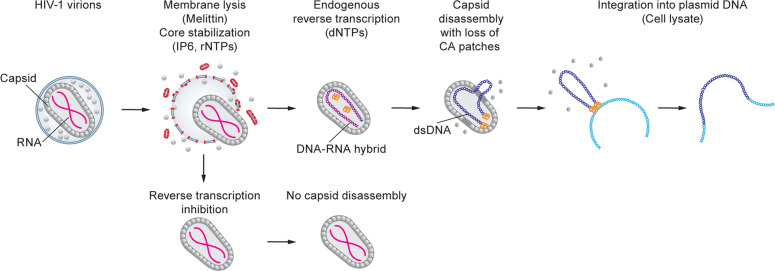
HIV-1 virions are lysed gently with melittin in the presence of IP6 and rNTPs to stabilize the viral cores. The addition of dNTPs initiates endogenous reverse transcription (ERT), which first copies viral RNA to form minus-strand DNA, and subsequently copies the minus-strand DNA to form double-stranded DNA (dsDNA). The dsDNA, which is rigid compared to genomic RNA or single-stranded DNA, creates internal pressure and initiates capsid disassembly, with localized loss of capsid (CA) patches. The viral dsDNA can integrate into a target plasmid DNA, which requires capsid disassembly and the addition of cell lysate. When reverse transcription is inhibited, very few disassembled capsids are observed, indicating that reverse transcription is required for capsid disassembly

To develop an efficient endogenous reverse transcription (ERT) assay, Christensen and colleagues used purified virions, gently lysed them with a pore-forming melittin peptide from honey bee venom, and empirically optimized buffer, salt, deoxynucleotide triphosphates (dNTPs), rNTPs, inositol hexokinase (IP6), and cell extracts.^1^ One of the key differences between this study and previous efforts to develop efficient ERT assays was the addition of IP6, a highly negatively charged compound that was recently shown to bind to the central pore of capsid hexamers and stabilize the HIV-1 capsids.^2^ Careful quantitative PCR analysis of reverse transcription products indicated that in ∼50% of the viral cores reverse transcription progressed to a late stage called plus-strand strong-stop DNA transfer, confirming that the conditions of the ERT assay supported efficient reverse transcription (Fig. 1).

Next, the authors examined how capsid stability affects reverse transcription by determining how capsid mutants that increase capsid stability, moderately decrease stability, or severely decrease stability affect reverse transcription efficiency. In addition, they determined the effect of IP6 concentrations on the efficiency of reverse transcription for wild-type, as well as the mutant capsids. The results showed that IP6 concentrations close to the estimated cellular concentrations yielded peak reverse transcription products for wild-type virions, suggesting that there is optimal capsid stability for supporting reverse transcription. The optimal IP6 concentrations were lower for hyperstable capsids and higher for less stable capsids, providing additional evidence that optimal capsid stability is important for efficient reverse transcription. Furthermore, treatment of capsids with capsid-binding inhibitor GS-CA1 disrupted capsids and inhibited reverse transcription, indicating that maintaining intact capsids is critical for efficient DNA synthesis.

Electron cryotomography (ECT) and subtomogram lattice mapping were employed to visualize individual cores at the subunit level. Under the conditions of the ERT assay, the morphologies of the individual capsids varied greatly at a time when most of the late reverse transcription products had been synthesized (8–10 h). While most cores (56%) remained largely intact, a significant proportion of cores (36%) had morphologies spanning a continuum from partially disassembled to almost completely disassembled capsid shells. The partially disassembled capsids were often missing local areas of CA hexamers, indicating that patches of CA hexamers were lost without complete disassembly of the capsid. In many of the partially disassembled capsids, the authors often observed viral DNA strands exiting and looping back into the capsid. The DNA loops extruding out of the partially disassembled capsids were estimated to be quite short, ranging from 0.5 to 1.5 kb, suggesting that most of the 9.7-kb DNA remained tightly packed inside the remaining capsid shell. In the absence of dNTPs, a majority (82%) of the capsids remained largely intact, indicating that reverse transcription promotes capsid disassembly. It remains unclear whether the capsid disassembly initiates only after completion of viral DNA synthesis, whether the partially disassembled capsids represent abortive reverse transcription, or whether reverse transcription can be completed after the capsid is partially disassembled. Nevertheless, one can envision that the authors have captured the uncoating process, with each partially disassembled capsid potentially representing a different stage of uncoating on the path toward complete capsid disassembly. Consistent with a previously proposed model, the authors suggest that the formation of dsDNA, which is relatively rigid compared to RNA, increases internal capsid pressure, resulting in local disruption of the capsid.^3^

At least some of the reverse-transcribed dsDNA synthesized in the ERT assay could integrate into a plasmid DNA target using the virion-incorporated IN. The integration was dependent on the completion of viral DNA synthesis and sensitivity to IN inhibitors. The authors also documented concerted integration, and nearly all of the events involved the expected 5′ target site duplication. Integration also required capsid disassembly as previously reported for HIV-1 capsids in the nuclei of infected cells.^4^ Strikingly, integration required the addition of cell lysate, indicating that one or more host factor(s) is needed to facilitate the reaction.

These studies describe efficient in vitro reconstitution of HIV-1 reverse transcription and integration. The results show that reverse transcription evolved to be an efficient process, that an intact capsid is essential for viral genome replication, and that capsid disassembly is a requirement for integration. Historically, it has not been possible to address these questions in infected cells because most viral cores that enter cells do not lead to productive infection and it has been difficult to quantify the amount of CA associated with the infectious viral cores. Our recent studies show that the viral cores remain intact or nearly intact in the nuclei of infected cells and undergo a rapid uncoating event (<20 min) near the site of integration shortly before integration.^4^ The results of these robust cell-free assays, and those from cell-based assays by us and others have gratifyingly converged to show that reverse transcription primarily occurs in the context of an intact viral core.^4,5^ It is anticipated that the cell-free ERT assays described by Christensen et al. will yield many insights into the early stage of HIV-1 replication for years to come.^1^
